# A Supreme National Health Authority

**Published:** 1907-10-05

**Authors:** 


### A Supreme National Health Authority.

Such is the title of a circular issued by the Chairman of the Sanitary Committee and the Medical Officer of Health for Newcastle-on-Tyne, which has been sent to the Public Health Committees of the County Councils, County Boroughs, Municipal Boroughs, and Port Sanitary Authorities of England and Wales, inviting them on behalf of a provisional Executive Committee to a Conference to be held in London on the 13th, 14th, or 15th November next. The object of the Conference, as set forth in the circular, is somewhat ambiguous. It is for "the purpose of considering the question of the establishment of a Permanent Union of the Sanitary Authorities of the Kingdom into a *Supreme National Health Authority.*" That a Supreme National Health Authority should be established is a tenet of our faith which we have consistently maintained, but in what manner a permanent union of the sanitary authorities of the kingdom can constitute this authority we have yet to learn. "The proposal," says the circular, "has been favourably commented on in the Press, both professional and lay," and we should have been glad to have joined in the general approval, if only we could have felt sure what precisely it was we were approving.

The combination of sanitary authorities into "one large permanent union" strikes us as innocent enough in itself, but if such a body is to become a Supreme National Health Authority, we pause aghast at the Frankenstein possibilities which we may be helping to call into being. This monster will be neither a government department nor a mother nor daughter of Parliaments. Whence it is to derive its authority we forbear to ask; but we shudder at the dreadful possibilities of this supreme incorporation of all the Bumbledoms attempting to secure uniformity of action among the diverse and heterogeneous interests of which it will be composed.

But perhaps "supreme authority" is merely a figure of speech, the reactionary hyperbole of those who, having groaned in spirit at the plentiful lack of co-ordination in matters affecting the public health, have seen in the movement they have originated a short road to the realisation of their hopes. We are too deeply impressed with the serious need for a central really supreme authority on health to share in the illusion.

If the union of local health authorities will expedite the formation of a Government Department of Health, with a Cabinet Minister as the supreme health authority, it will deserve well and command the support of all who have the great interests of public health at heart. But such a union must not mistake its mission and suppose itself a supreme or in any sense an authority at all. There is ample scope for its activities in the less ambitious field of mutual conference. In such an arena there will be unquestionable profit to the municipalities which are represented, and we can well conceive a wholesome leavening of the Health Committee mind when it contemplates a wider area than the several wards which too often monopolise its constricted attention.

Yet even in this respect we fear our qualified approval of the Union is further tempered by the fact that already there are annual conferences or congresses of public health organised by two separate Royal institutes, and numerous congresses on particular items of public health interest convened independently as occasion seems to demand. Further, to multiply these organisations will require to be justified by more than a confused purpose ambiguously stated. And just because congresses play so useful a part in municipal life, it is important not to weary by a too frequent and tiresome repetition. We should be sorry were a check to be put to the useful work steadily being accomplished by the already too frequently recurring health congresses, but this is almost certain to follow should it be felt that their recurrence was inordinate, or their cost in time and money disproportionate to the results they can legitimately claim.

There is so much local work for the health authorities to do; there is so much central work which must not be done by the local authorities even in combination; and there is such need for a properly constituted central authority, supreme in matters of health, that we have felt constrained to criticise rather than praise the well-meaning movement that emanates from the northern city.

